# Supplementary material for: Gene editing of SAMHD1 in macrophage-like cells reveals complex relationships between SAMHD1 phospho-regulation, HIV-1 restriction, and cellular dNTP levels
Source: mBio. 2023 Oct 6;14(5):e02252-23. doi: 10.1128/mbio.02252-23 (PMC10653793; doi:10.1128/mbio.02252-23)
Supplement: Fig. S1 — Validation of CRISPR/Cas9 mediated mutagenesis of SAMHD1 catalytic core residues. [file mbio.02252-23-s0001.pdf]

Figure S1

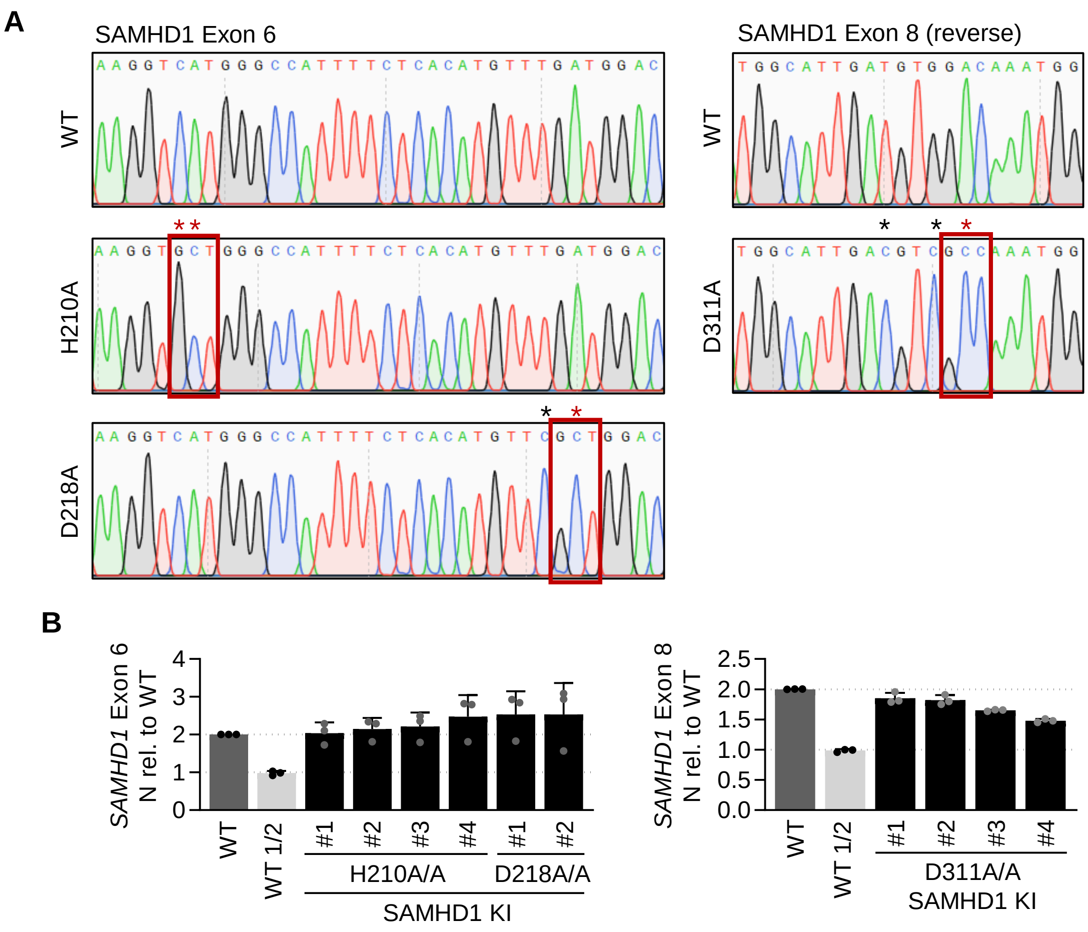

Figure S1: Validation of CRISPR/Cas9 mediated mutagenesis of SAMHD1 catalytic core residues. (A) Representative sections of Sanger sequencing traces obtained from genomic *SAMHD1* exon 6 and 8. Base triplets corresponding to modified amino acids are highlighted. Asterisk indicate coding and silent mutations introduced. At least two independent sequencing runs were performed per clone. (B) Quantitative genomic PCR for *SAMHD1* exon 6 and 8 against reference gene *TERT* was performed and $2^{-\Delta ct}$ value obtained from SAMHD1 KI clones normalized to WT. As a control, half of the WT (WT 1/2) DNA was inoculated and Δct of SAMHD1 calculated against ct of TERT which was obtained in the WT with normal DNA amount. Bar graphs indicate mean of experiments, dots individual biological replicates. Error bars correspond to standard deviation (n = 3).
